# Clinical and safety outcomes of BeEAM (Bendamustine, Etoposide, Cytarabine, Melphalan) versus CEM (Carboplatin, Etoposide, Melphalan) in lymphoma patients as a conditioning regimen before autologous hematopoietic cell transplantation

**DOI:** 10.1186/s12885-024-12694-9

**Published:** 2024-08-13

**Authors:** Mohamed A. Eltelbanei, Noha A. El-Bassiouny, Mahmoud Salah Abdalla, Mohamed Khalaf, Rehab H. Werida

**Affiliations:** 1 Senior Clinical Pharmacist in Hematology, Oncology, and Stem Cell Transplantation at International Medical Center (IMC) Hospital, Clinical pharmacy & pharmacy practice master candidate at Faculty of pharmacy, Damanhour University, Cairo, Egypt; 2 Clinical Pharmacy & Pharmacy Practice Department, Faculty of Pharmacy, Damanhour University, Damanhour, 22514 Egypt; 3 Hematology& BMT Department at International Medical Center (IMC) Hospital, Cairo, Egypt; 4 Consultant of Hematology & BMT Department at Maadi Military Hospital, Cairo, Egypt

**Keywords:** Autologous transplantation, BeEAM protocol, CEM protocol, DMSO, Lymphoma, Non-cryopreservation

## Abstract

**Background:**

Autologous stem cell transplantation (ASCT) is a pivotal treatment for lymphoma patients. The BeEAM regimen (Bendamustine, Etoposide, Cytarabine, Melphalan) traditionally relies on cryopreservation, whereas the CEM regimen (Carboplatin, Etoposide, Melphalan) has been optimized for short-duration administration without the need for cryopreservation. This study rigorously compares the clinical and safety profiles of the BeEAM and CEM regimens.

**Methods:**

A controlled, randomized clinical trial was conducted with 58 lymphoma patients undergoing ASCT at the International Medical Center (IMC) in Cairo, Egypt. Patients were randomly assigned to either the BeEAM (*n* = 29) or CEM (*n* = 29) regimen, with an 18-month follow-up period. Clinical and safety outcomes were meticulously compared, focusing on time to engraftment for neutrophils and platelets, side effects, length of hospitalization, transplant-related mortality (TRM), and survival rates.

**Results:**

The findings demonstrate a significant advantage for the CEM regimen. Neutrophil recovery was markedly faster in the CEM group, averaging 8.5 days compared to 14.5 days in the BeEAM group (*p* < 0.0001). Platelet recovery was similarly expedited, with 11 days in the CEM group versus 23 days in the BeEAM group (*p* < 0.0001). Hospitalization duration was substantially shorter for CEM patients, averaging 18.5 days compared to 30 days for those on BeEAM (*p* < 0.0001).

Furthermore, overall survival (OS) was significantly higher in the CEM group at 96.55% (95% CI: 84.91–99.44%) compared to 79.31% (95% CI: 63.11–89.75%) in the BeEAM group (*p* = 0.049). Progression-free survival (PFS) was also notably superior in the CEM group, at 86.21% (95% CI: 86.14–86.28%) versus 62.07% (95% CI: 61.94–62.20%) in the BeEAM group (*p* = 0.036).

**Conclusion:**

The CEM regimen might demonstrate superiority over the BeEAM regimen, with faster neutrophil and platelet recovery, reduced hospitalization time, and significantly improved overall and progression-free survival rates. Future studies with longer duration and larger sample sizes are warranted.

**Trial registration:**

This study is registered on ClinicalTrials.gov under the registration number NCT05813132 (https://clinicaltrials.gov/ct2/show/NCT05813132).

(The first submitted registration date: is March 16, 2023).

## Highlights

- This trial compares BeEAM (depends on cryopreservation) versus CEM protocol (doesn’t need cryopreservation) as a conditioning regimen before ASCT.
- The CEM regimen can be superior to the BeEAM regimen regarding neutrophil and platelet recovery, shorter hospital stays, fewer side effects, TRM, and relapse.
- Moreover, the CEM regimen showed an add-on value on OS and PFS.

## Background

Lymphoma is a malignancy that affects the lymphatic system, classified into two main categories: Hodgkin lymphoma (HL) and non-Hodgkin lymphoma (NHL), based on the presence of Reed-Sternberg cells observed during pathological examination [1, 2]. NHL comprises subtypes of lymphoid malignancies, each characterized by specific pathological features. The World Health Organization (WHO) Classification of Hematolymphoid Tumors is widely recognized as the global standard for diagnosing these tumors [3].

Treatment strategies for lymphoma vary depending on the disease subtype, stage, and individual patient factors. One of the potentially effective treatments is hematopoietic cell transplantation (HCT) [4].

HCT is an infusion of hematopoietic cells into a patient's body following cytotoxic conditioning regimens. These regimens employ potent cytotoxic agents to eradicate the disease and create an optimal environment for successful transplantation. HCT is regarded as a potentially curative treatment for certain hematologic malignancies [4].

HCT is categorized into two primary types based on the source of the hematopoietic cells: autologous and allogeneic.

Autologous stem cell transplantation (ASCT) is indicated for various lymphoma subtypes, reflecting its critical role in modern hematologic oncology. It is employed as upfront consolidation therapy for first remission in mantle cell lymphoma (MCL) [5] and is also a vital treatment option for primary CNS lymphoma (PCNSL) [6, 7], ASCT is crucial for managing high-risk diffuse large B-cell lymphoma (DLBCL) [8], and T-cell lymphoma [9], Furthermore, it is indispensable for relapsed or refractory (RR) lymphomas of other subtypes, which remain challenging to treat despite their initial responsiveness to chemotherapy [10, 11].

Conventional salvage treatments often do not give good results for patients. However, high-dose chemotherapy (HDCT) followed by stem cell rescue has significantly improved disease-free survival (DFS) for patients who have relapsed but responded well to chemotherapy. As a result, autologous stem cell transplantation (ASCT) following HDCT has become the preferred approach for treating these types of lymphomas [12, 13].

One of the most established conditioning regimens before ASCT is BEAM (BCNU/Carmustine, Etoposide, Cytarabine, and Melphalan). However, toxicity issues and the limited availability of carmustine have necessitated its replacement with other agents in various protocols. Substitutions include Bendamustine in the BeEAM protocol, thiotepa in the TEAM protocol, and CCNU (Lomustine) in the LEAM/CEAM protocols [14–16].

Peripheral blood stem cells (PBSCs) can be stored at 4°C for up to 72 h, with certain studies indicating potential viability extension up to 7 days. However, the viability of PBSCs diminishes gradually from 98 to 82% over time [17, 18].

The BeEAM regimen traditionally entails a 7-day administration schedule, necessitating the cryopreservation of stem cells. However, this cryopreservation process poses several challenges, including diminished cell viability post-thawing, potential adverse reactions in patients stemming from cryoprotectants like DMSO (Dimethyl Sulfoxide), and an increase in transplantation costs [17, 19].

Numerous clinical studies have directly compared the outcomes of conditioning regimens that utilize frozen stem cells versus those employing non-frozen, fresh stem cell preparations. This research has consistently demonstrated the benefits of using non-frozen stem cells. Patients conditioned with fresh stem cells have shown improved engraftment rates, reduced toxicity profiles, and faster hematopoietic recovery than those receiving frozen stem cell products [18, 20, 21]. Moreover, the limited availability of DMSO in low or middle-income countries and the potential adverse effects associated with its use, such as cardiac complications [22] further support the use of non-frozen regimens.

An alternative conditioning regimen known as CEM (Carboplatin, Etoposide, and Melphalan) has been explored as a cryopreservation-free approach. This regimen has been effectively utilized in lymphoma patients at Maadi Military Hospital in Cairo, Egypt, as communicated by Dr. Mohamed Khalaf. The CEM protocol was also employed as a high-dose chemotherapy conditioning strategy before ASCT for neuroblastoma patients [23].

While most HDCT conditioning regimens for lymphomas do not typically incorporate platinum compounds, a pilot study sought to evaluate the effectiveness of an HDCT regimen combining carboplatin with two other commonly used drugs, melphalan and etoposide [24]. The rationale for this approach was the potential of cisplatin-based salvage chemotherapy, particularly in combination with alkylating agents and podophyllotoxins, to demonstrate efficacy in lymphoma [25, 26].

Similarly, the CEM regimen has exhibited efficacy in the treatment of non-hematological malignancies, such as breast and ovarian cancers [27], germ cell tumors (GCTs) [28], and neuroblastoma [29]. These findings suggest the versatility of the CEM conditioning strategy across various oncologic contexts.

The primary objective of the current study is to compare the safety and toxicity profiles, engraftment kinetics for neutrophils and platelets, and hospital length of stay between the CEM and the more traditional BeEAM conditioning regimens in the setting of ASCT for lymphoma patients. The secondary objectives are to assess transplant-related mortality (TRM) and evaluate improvements in survival outcomes, including overall survival (OS) and progression-free survival (PFS).

## Methods

### Study design

This study is an open-label, prospective, parallel randomized controlled trial conducted at the International Medical Center (IMC) in Cairo, Egypt, from November 2022 to December 2023 (The data were collected retrospectively from electronic hospital records from July to November 2022 and prospectively from November 2022 to December 2023). Institutional Review Board approval was obtained from the Damanhour University ethical committee (approval number IRB no. 1122PP58) before the commencement of the study. Following the principles outlined in the Declaration of Helsinki, all patients provided written informed consent before participation. This study is registered on ClinicalTrials.gov under registration number NCT05813132 (https://clinicaltrials.gov/ct2/show/NCT05813132).

Patients were randomly assigned in equal numbers, in a 1:1 ratio, to two groups: one received the BeEAM regimen and the other received the CEM regimen. The random assignment was conducted using a simple randomization method, facilitated by a web-based randomization system (https://www.randomizer.org/) to generate random numbers.

### Patients

Patients were included in this study if they met the following inclusion criteria: diagnosed lymphoma in first remission (mantle cell lymphoma [MCL], high-risk diffuse large B-cell lymphoma [DLBCL], T-cell lymphoma, or relapsed/refractory [RR] lymphomas of other subtypes), aged between 18 and 65 years old, with no prior stem cell interventions (i.e., ASCT-naïve), and eligible for ASCT. Exclusion criteria included the presence of CNS lymphoma, lymphoma accompanied by a solid tumor, pregnancy, breastfeeding, other comorbid diseases that prevent ASCT, CD34 + stem cell count below 2 × 10^6^ cells [30, 31], or unwillingness to participate in the study.

The disease status of each patient, categorized as either complete remission (CR) or partial remission (PR), was assessed using 18F-Fluorodeoxyglucose Positron Emission Tomography-computed Tomography (18F-FDG PET-CT) before transplantation.

### Transplant procedures

Participants eligible for ASCT and meeting the inclusion criteria began mobilization of PBSC four days before harvesting. This was achieved using Granulocyte Colony-Stimulating Factor (G-CSF): filgrastim (10 μg/kg/day SC) with or without plerixafor (mozobil®) (0.24 mg/kg SC, maximum dose 40 mg), or through chemo-mobilization based on the patient's condition [32].

Plerixafor was administered to patients predicted to be poor mobilizers, identified by several risk factors, a peripheral CD34 + level below 20 cells/µL on the fourth day of mobilization, or an inadequate hematopoietic stem cell yield on the first day of apheresis [30, 31, 33].

Following mobilization, leukapheresis or stem cell collection was performed on the fifth and sixth days of G-CSF administration. Each leukapheresis session was limited to a maximum of five hours, in this study, each patient underwent only two sessions [34]. Flow cytometry was used to evaluate the CD34 + cell count to ensure an optimal number of cells were collected.

Post-leukapheresis, patients were randomly assigned to either the BeEAM or CEM group using a 1:1 allocation ratio. The randomization was facilitated by a web-based system at https://www.randomizer.org.

For the CEM group, freshly collected cells were stored at 4 °C for 48–72 h and were planned to be infused 24 h after the last chemotherapy dose. For the BeEAM group, freshly collected cells underwent cryopreservation. PBSCs were diluted with autologous plasma or a commercial resuspension medium, and the cell concentration was increased through volume depletion to reduce the number of cryo-stored bags required. The final product was mixed with 5–10% dimethyl sulfoxide (DMSO) as a cryoprotectant and 0.05–0.25 mL of Anticoagulant Citrate Dextrose Solution (ACD-A) stabilizer solution per milliliter of transplant. Cells were frozen at a controlled rate of 1–2 °C per minute and stored in vapour phase nitrogen at temperatures ≤  − 140 °C. During ASCT, cryopreserved bags were thawed and PBSCs were reinfused within a maximum of 10–20 min [35].

### Conditioning regimens and supportive care

The doses of the two protocols are illustrated in Table 1. Several important considerations related to the CEM regimen must be highlighted. To achieve the maximum myeloablative dose of carboplatin, adhering to the recommendations of the American Society of Clinical Oncology (ASCO) we did not cap the body surface area (BSA) at 2 mg/m^2^, [36]. The American Society for Blood and Marrow Transplantation (ASBMT) also recommended calculating BSA for carboplatin based on total body weight (TBW) [37], ensuring it does not exceed the maximum tolerated dose (MTD) [38]. The etoposide dose was maintained at 30 mg/kg to achieve myeloablation without surpassing the MTD [39]. ASBMT guidelines specify that etoposide should be calculated using the formula [IBW (ideal body weight) + 0.25(TBW—IBW)] [37].

**Table 1:** Conditioning chemotherapy regimen

Conditioning chemotherapy regimen	Doses	Days
BeEAM [14]	Bendamustine dose of 160 mg/m^2^/day IV	-7 and -6
	Etoposide 200 mg/m^2^ /day BID IV	-5 to –2
	Cytarabine 200 mg/m^2^ /day IV BID	-5 to -2
	Melphalan 140 mg/m^2^ IV	-1
CEM [23]	Carboplatin 1000 mg/m^2^ IV total dose divided over the two days	Over 36-37 h
	Etoposide 30 mg/kg IV total dose divided over the two days	
	Melphalan 140 mg/m^2^ total dose divided into over the days	

**Fig. 1:**
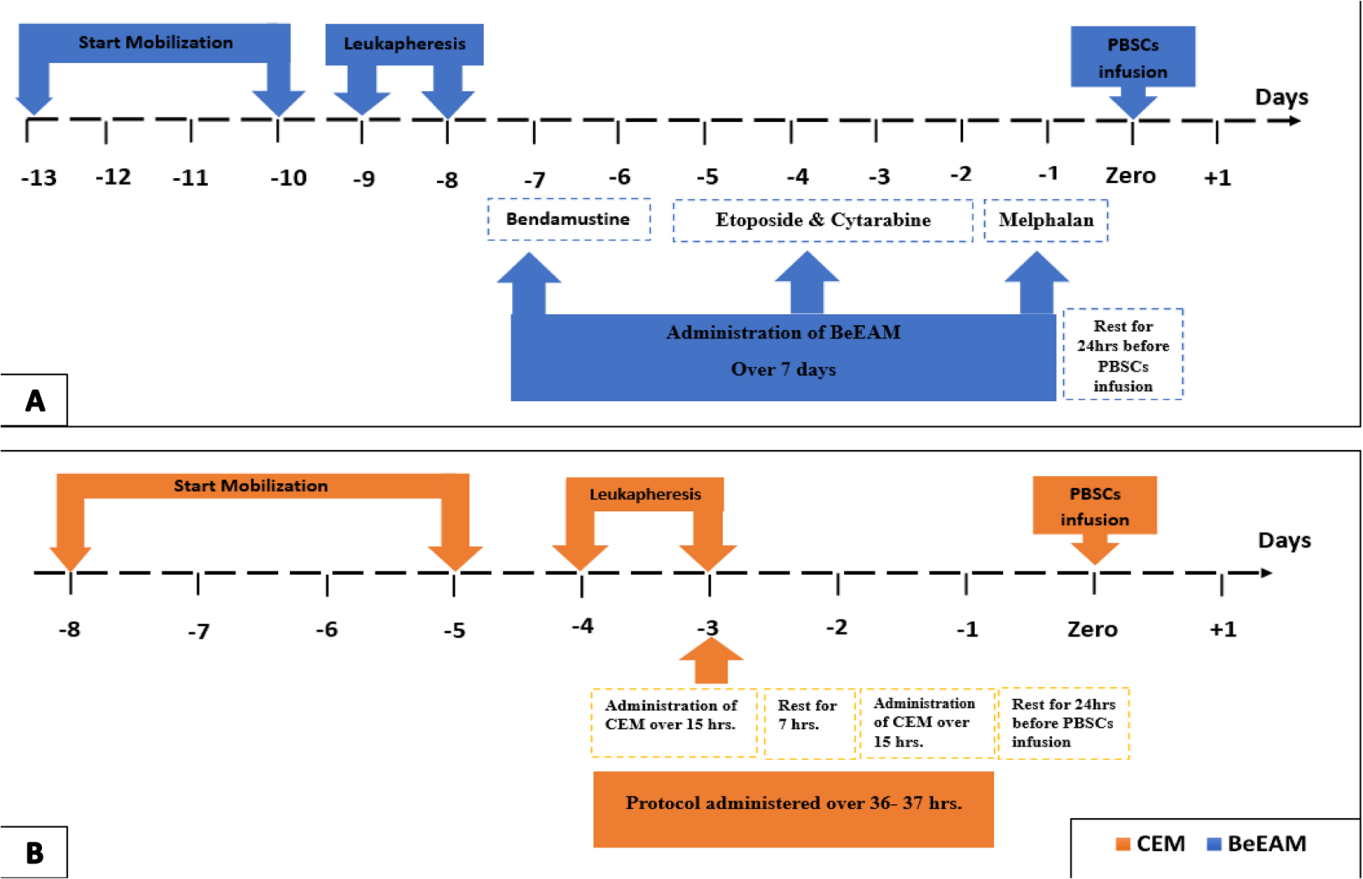
Illustration of transplantation process, regimens administration. **A** BeEAM protocol. **B** CEM protocol

To preserve the viability of the peripheral blood stem cells (PBSCs) without cryopreservation in the CEM regimen, the protocol was administered over 36 h: 11–12 h on the first day according to the drug doses, followed by a 7-h rest period, and then 13–15 h on the second day. There was a 24-h rest period between the last chemotherapy dose and the stem cell infusion for both protocols. The transplantation process and regimen administration are illustrated in Fig. 1. Each patient received filgrastim (G-CSF) at a dose of 5 μg/kg, starting from day + 6 post-ASCT until the absolute neutrophil count reached 0.5 × 10^9^/L for three consecutive days, at which point the G-CSF was discontinued. Platelet or red blood cell transfusions were provided when the platelet count fell below 10 × 10^9^/L or the haemoglobin level dropped below 8 g/dL, respectively.

ASCT presents an intermediate risk for infections, as per National Comprehensive Cancer Network (NCCN) guidelines. Therefore, all patients received prophylactic medications from day zero until recovery, including oral acyclovir 400 mg BID for antiviral protection, oral fluconazole 400 mg OD for antifungal prevention, and oral levofloxacin 500 mg OD for antibacterial defense. Hyperuricemia prophylaxis for tumor lysis syndrome was initiated at the start of conditioning and continued until day 0, using oral allopurinol at 300 mg/m^2^ in three divided daily doses concurrent with hydration. Prophylaxis for hepatic veno-occlusive disease included daily enoxaparin administered subcutaneously until platelet counts dropped below 50 × 10^9^/L and ursodeoxycholic acid 13–15 mg/kg/day, divided into two to four doses [40–43].

### Assessment and follow-up

Adverse effects and toxicities were systematically assessed using the Common Terminology Criteria for Adverse Events (CTCAE) version 5.0. Parameters monitored included nausea, vomiting, diarrhea, febrile neutropenia, hypokalemia, renal and hepatic function, and mucositis. Engraftment was evaluated with precise criteria: neutrophil counts exceeding 0.5 × 10^9^/L for three consecutive days, and platelets surpassing 20 × 10^9^/L. For this study, we adopted a more stringent threshold of 50 × 10^9^/L for platelet recovery. The criteria for defining engraftment were aligned with the guidelines established by the European Society for Blood and Marrow Transplantation (EBMT) [44].

Transplant-related mortality (TRM), defined as death within 100 days post-transplantation due to severe complications, was carefully monitored. Patients were followed for 18 months, with 18F-FDG PET-CT scans every 6 months to monitor for relapse. The primary outcomes included overall survival (OS), which encompassed all-cause mortality during the study period; progression-free survival (PFS), defined as the time to death or relapse; and disease-free survival (DFS), specifically tracking relapse events for each treatment regimen.

### Statistical analysis

The sample size for this study was determined based on effect sizes reported in previous studies that utilized similar sample sizes and conditioning regimens [45, 46]. The post-hoc sample size calculation was performed using G*Power software version 3.1.0 (Institut für Experimentelle Psychologie, Heinrich Heine Universität, Düsseldorf, Germany). A total sample size of 58 patients was estimated to provide a power of 83.2% to detect a medium to large effect size of 0.80 in the measured outcomes. Recognizing the small sample size and the prospective nature of our study, we selected this method to ensure accurate and reliable results. The analysis indicated that a total sample size of 58 patients (29 in each group) would provide a power of 83.2% to detect a medium to large effect size of 0.80 in the measured outcomes.

All statistical analyses were conducted using SPSS Statistics version 26. The Kolmogorov–Smirnov and Shapiro–Wilk tests were applied to assess the normality of data. For normally distributed data, a two-sample independent t-test was conducted, with adjustments made for equal or unequal variances as appropriate, to compare the two study groups. The Mann–Whitney U test was utilized for data that did not follow a normal distribution.

For categorical variables, Pearson’s Chi-square or Fisher’s exact tests were utilized to identify differences in the severity grades of adverse effects, such as vomiting, diarrhea, febrile neutropenia, hepatotoxicity, and renal toxicity, between the two groups at each time point.

The Kaplan–Meier method was used to estimate (OS), (PFS), (DFS). Comparisons between the study groups were conducted using the log-rank test. All analyses were performed on an intention-to-treat (ITT) basis, with a significance level set at *P* < 0.05 for all reported results.

## Results

### Patient characteristics

**Fig. 2:**
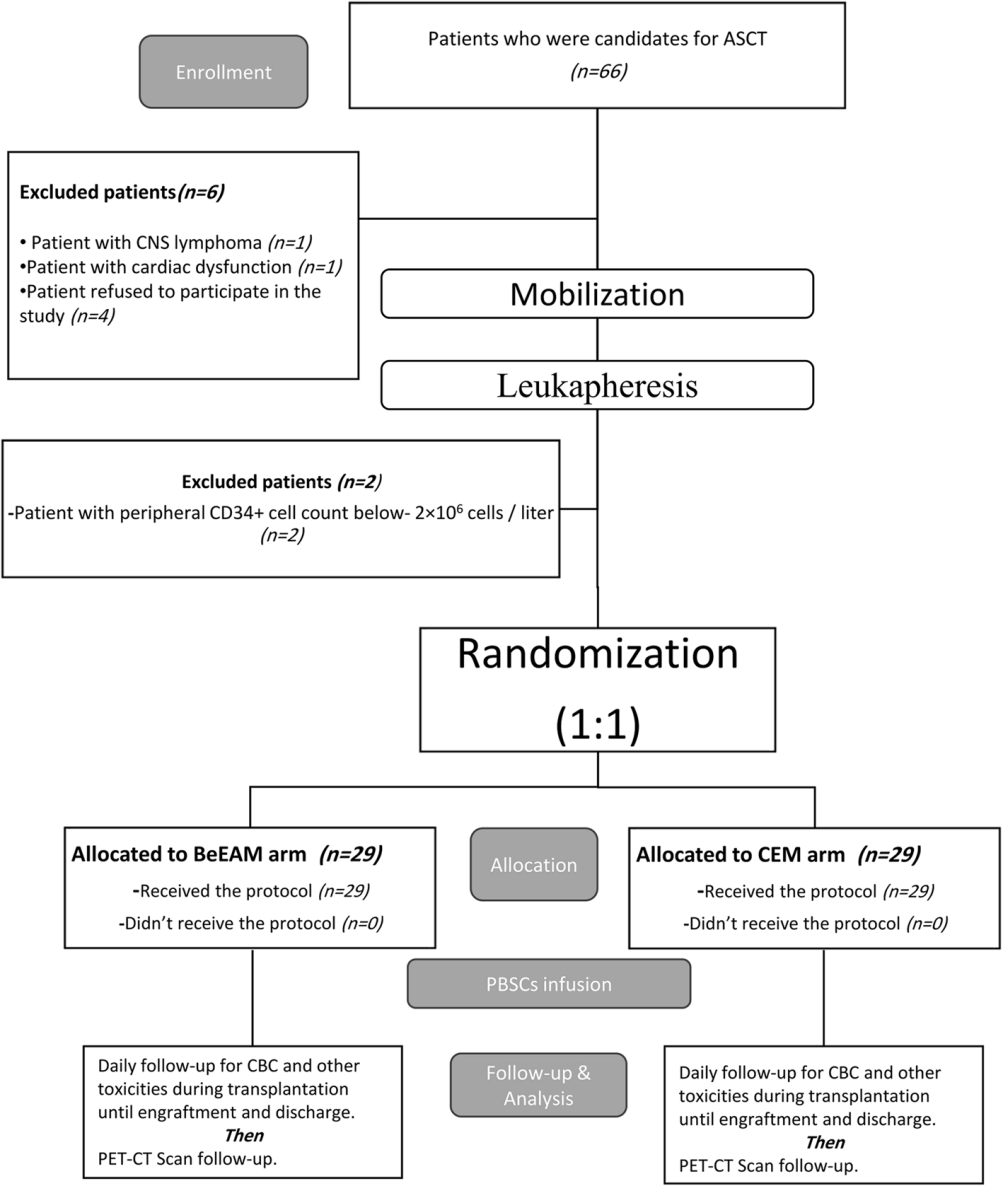
The Consolidated Standards of Reporting Trials (CONSORT) diagram. PBSCs: peripheral blood stem cells

Sixty-six patients who were candidates for ASCT were screened for eligibility. Fifty-eight patients met the inclusion criteria and were deemed eligible, while eight were excluded due to meeting one or more exclusion criteria. The eligible 58 patients were randomly assigned in a 1:1 ratio into two groups: one group received the BeEAM protocol, and the other received the CEM protocol. The study flow is summarized in the CONSORT diagram (Fig. 2). Baseline characteristics of the patients are presented in Table 2, with no statistically significant differences observed between the two groups.

**Table 2:** Baseline characteristics data of the studied patients

Characteristics:	BeEAM (*n* = 29)	CEM (*n* = 29)	Risk assessment. (OR)	*P-Value*
Age: (mean ± SD) years^*^	39.26 ± 11.71	40.43 ± 11.50	1(0.95–1.05)	0.95
Gender* n* (%):^**^				
Male no. (%):	20 (69%)	16 (55.2%)	1,8 (0.62–5.29)	
				0.28
Female no. (%):	9 (31%)	13 (44.8%)		
Diagnosis:^**^				
***RHL no (%):***	15 (51.7%)	13 (44.8%)	1.32(0.47–3.70)	0.28
***RNHL no (%):***	14 (48.2%)	16 (55.1%)		
RDLBCL no (%):	11 (37.9%)	12 (41.4%)	0.86(0.30–2.48)	0.72
Mantle cell lymphoma no (%):	1 (3.4%)	0	2(1.56–2.65)	1
ALCL no (%):	2 (6.9%)	2 (6.9%)	1(0.13–7.62)	1
Burkitt lymphoma no (%):	0	1 (3.4%)	2(1.56–2.65)	1
Follicular lymphoma no (%):	0	1 (3.4%)	2(1.56–2.65)	1
ECOG-PS Score:^**^				
Zero no:	27 (93.1%)	28 (96.5%)	0.48(0.04–5.63)	0.35
One no:	2 (6.9%)	1 (3.5%)	2.07(0.18–24.23)	0.35
**Previous therapies** (included protocols for newly diagnosed and relapsed patients)^**^:				
***Hodgkin lymphoma patients***				
ABVD	13 (44.8%)	13 (44.8%)	1(0.35–2.82)	1
BEACOPP	2 (6.9%)	0	2.07(1.58–2.72)	0.49
DHAP	1 (3.5%)	0	2.04(1.56–2.65)	1
ESHAP	0	1 (3.5%)	2.04(1.56–2.65)	1
GDP	1 (3.5%)	1 (3.5%)	1(0.06–16.79)	1
ICE	4 (13.8%)	3 (10.3%)	1.29(0.28–6.81)	1
Brentuximab vedotin + Bendamustine	6 (20.7%)	5 (17.2%)	1.25(0.34–4.68)	1
Brentuximab vedotin + ICE	1 (3.5%)	1 (3.5%)	1(0.06–16.79)	1
***Non-Hodgkin lymphoma patients***				
RCHOP	12 (41.4%)	7 (24.1%)	2.22(0.72–6.85)	0.26
R-CHOP alternating with R-DHAP	1 (3.5%)	0	2.04(1.56–2.65)	1
R-Hyper-CVAD	0	1 (3.5%)	2.04(1.56–2.65)	1
Hyper-CVAD	0	1 (3.5%)	2.04(1.56–2.65)	1
R-GDP	4 (13.8%)	3 (10.3%)	1.39(2.82–6.80)	1
GDP	2 (6.9%)	1 (3.5%)	2.07(0.18–24.23)	1
R-ICE	4 (13.8%)	4 (13.8%)	1(0.23–4.45)	1
R-ESHAP	1 (3.5%)	1 (3.5%)	1(0.06–16.79)	1
R-DHAP	1 (3.5%)	0	2.04(1.56–2.65)	1
Obinutuzumab—Bendamustine	0	1 (3.5%)	2.04(1.56–2.65)	1
Rituximab—Bendamustine	0	2 (6.9%)	2.07(1.58–2.72)	0.49
Brentuximab vedotin + Bendamustine	2 (6.9%)	2 (6.9%)	1(0.13–7.62)	1
CHOEP	1 (3.5%)	0	2.04(1.56–2.65)	1
CHOP	1 (3.5%)	0	2.04(1.56–2.65)	1
GVD	1 (3.5%)	0	2.04(1.56–2.65)	1
Disease stage:^**^				
II	8 (27.6%)	5 (17.2%)	1.83(0.52–6.46)	0.89
III	9 (31%)	10 (34.5%)	0.85(0.29–2.56)	0.078
IV	12 (41.4%)	14 (48.3%)	0.76(0.27–2.14)	0.28
Remission status before ASCT:^**^				
CR no (%):	29 (100%)	26 (89.7%)	0.47(0.36–0.63)	0.075
PR no (%):	0	3 (10.3%)		
**CD34**^**+**^**count** × (10^6^ /kg), Median (Range)^a^	4.5 (1.53–50)	4.56 (0.5–23)	0.98(0.91–1.06)	0.73
Other Comorbidity:^**^				
HTN no (%):	4 (13.8%)	0	2.2(1.62–2.88)	0.11
DM no (%):	4 (13.8%)	3 (10.3%)	1.4 (0.28–6.83)	0.69

*RHL* Relapsed Hodgkin Lymphoma, *R-NHL* Relapsed non-Hodgkin Lymphoma, *RDLBCL* Relapsed Large B-cell lymphoma, *ALCL* Anaplastic large T-cell lymphoma, *ECOG-PS Score* Eastern Cooperative Oncology Group (ECOG) Performance Status, *CR* Complete Remission, *PR* Partial Remission, *HTN* Hypertension, *DM* Diabetic mellitus

^*^Data was represented as (mean ± Standard deviation) then an unpaired t-test for *p*-value and significance,

**Frequency and Categorical data represented as no (%): number (percentage) then use Fisher’s Exact Test or X2Chi-square test for *p*-value and significance,

^a^non-normal distribution data was represented as median (minimum–maximum) (range) then used Mann–Whitney-U-Test for *p*-value

### Engraftment & transfusion support

The median time for neutrophil recovery in the CEM group was 8.5 days, significantly shorter than the 14.5 days observed in the BeEAM group (*p* < 0.0001), indicating a faster Absolute Neutrophil Count (ANC) recovery with the CEM regimen. Similarly, the median duration for platelet recovery was significantly reduced with the CEM regimen (11 days) compared to the BeEAM regimen (23 days), also showing statistical significance (*p* < 0.0001). Due to the accelerated engraftment of neutrophils and platelets with the CEM protocol, the hospital length of stay was significantly shorter in the CEM group (18.5 days) compared to the BeEAM group (30 days), with a *p*-value of < 0.0001 (Table 3).

**Fig. 3:**
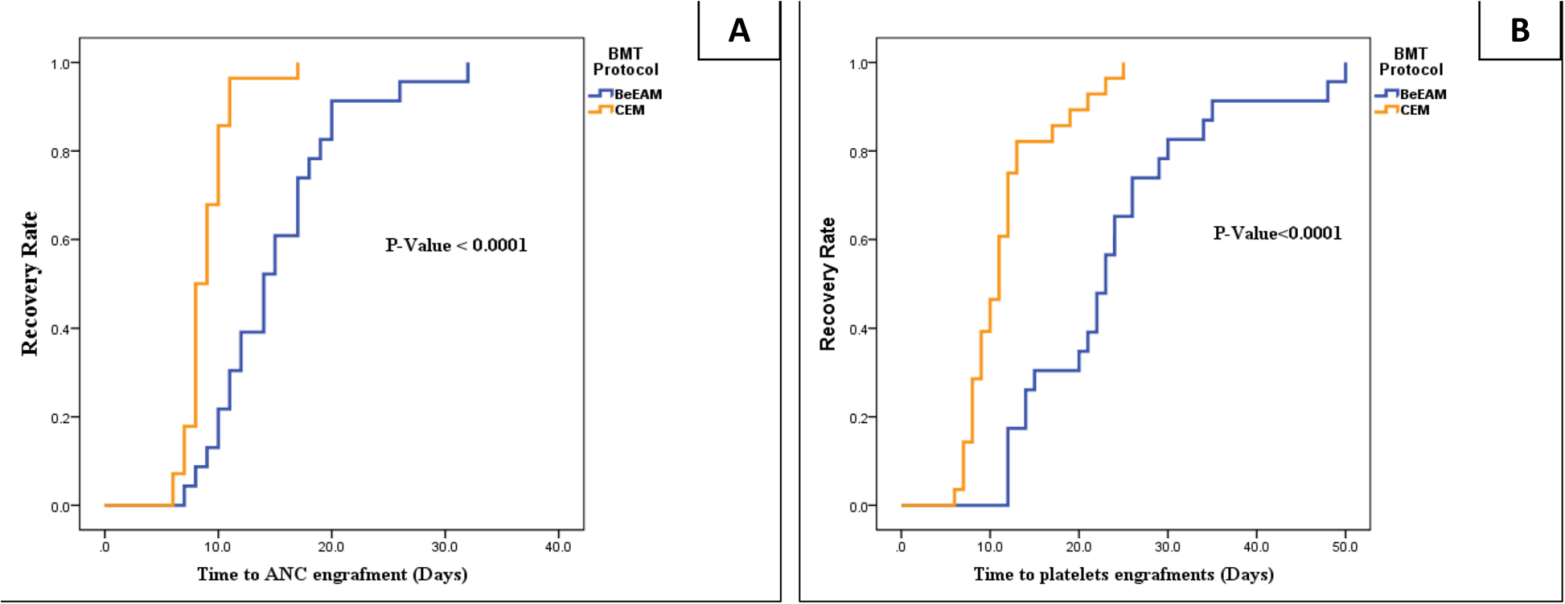
Recovery of neutrophils & platelets in both protocols. **A** Recovery rate of neutrophils for two protocols. **B** Recovery rate of platelets for two protocols. ANC; Absolute neutrophil count

Although the CEM regimen showed lower percentages of (TRM) and relapse compared to the BeEAM regimen, these differences did not reach statistical significance. In the BeEAM group, six patients (20.7%) experienced early mortality before engraftment, predominantly due to infection or septic shock, compared to only one patient (3.4%) in the CEM group. The results of engraftment data are summarized in Table 3. Table 4 shows that the frequency of blood and platelet transfusions was significantly higher in the BeEAM treatment group compared to the CEM regimen (*p* < 0.0001, the median time to blood transfusion was 1 day in the BeEAM group compared to 0 days in the CEM group. Similarly, the median time to platelet transfusion was 5.5 days in the BeEAM group compared to 2 days in the CEM group. These results indicate that the CEM regimen required significantly fewer blood and platelet transfusions than the BeEAM treatment. This suggests the CEM regimen may be associated with faster hematologic recovery and fewer transfusion support needs than the BeEAM approach as shown in Fig. 3.

**Table 3:** Hematopoietic engraftment data

	**BeEAM(*n* = 29)**	**CEM(*n* = 29)**	*P*-value
ANC recovery, Days^a^:	14.5(7–32)	8.5(6–17)	< 0.0001
Platelets recovery, Days^a^:	23(12–50)	11(6–25)	< 0.0001
Length of stay at the hospital^a^	30(23–43)	18.5(16–27)	< 0.0001
Transplant-related mortality (TRM)^**^	6 (20.7%)	1 (3.4%)	0.1
Relapse^**^	5 (21.7%)	3 (10.7%)	0.44

*ANC* Absolute neutrophil count

^a^non-normal distribution data was represented as median (minimum–maximum) (range) then used Mann–Whitney-U-Test for *p*-value

^**^Categorical data represented as no (%): number (percentage) then use X^2^Chi-square for *p*-value and significance. Bold fonts represent significant results

**Table 4:** Transfusion support therapy

	**BeEAM (*n* = 29)**	**CEM (*n* = 29)**	*P*-value
Blood transfusion, times^a^	1 (0–10)	0 (0–2)	< 0.0001
Platelets transfusion, times^a^	5.5 (1–12)	2 (0–7)	< 0.0001

The red blood cell transfusion threshold was less than 7 g\dl or 8 g\dl plus symptomatic patients such as cardiac problems. Bold fonts represent significant results. The platelets transfusion threshold was 10000 per microliter of blood or 20000 T0 30000 per microliter of blood if bleeding occurred

^a^Nonnormal distribution data was represented as median(minimum-maximum) (range) then used Mann-Whitney-U-Test for *p*-value. Bold fonts represent significant results

### Adverse effects and related toxicity

**Fig. 4:**
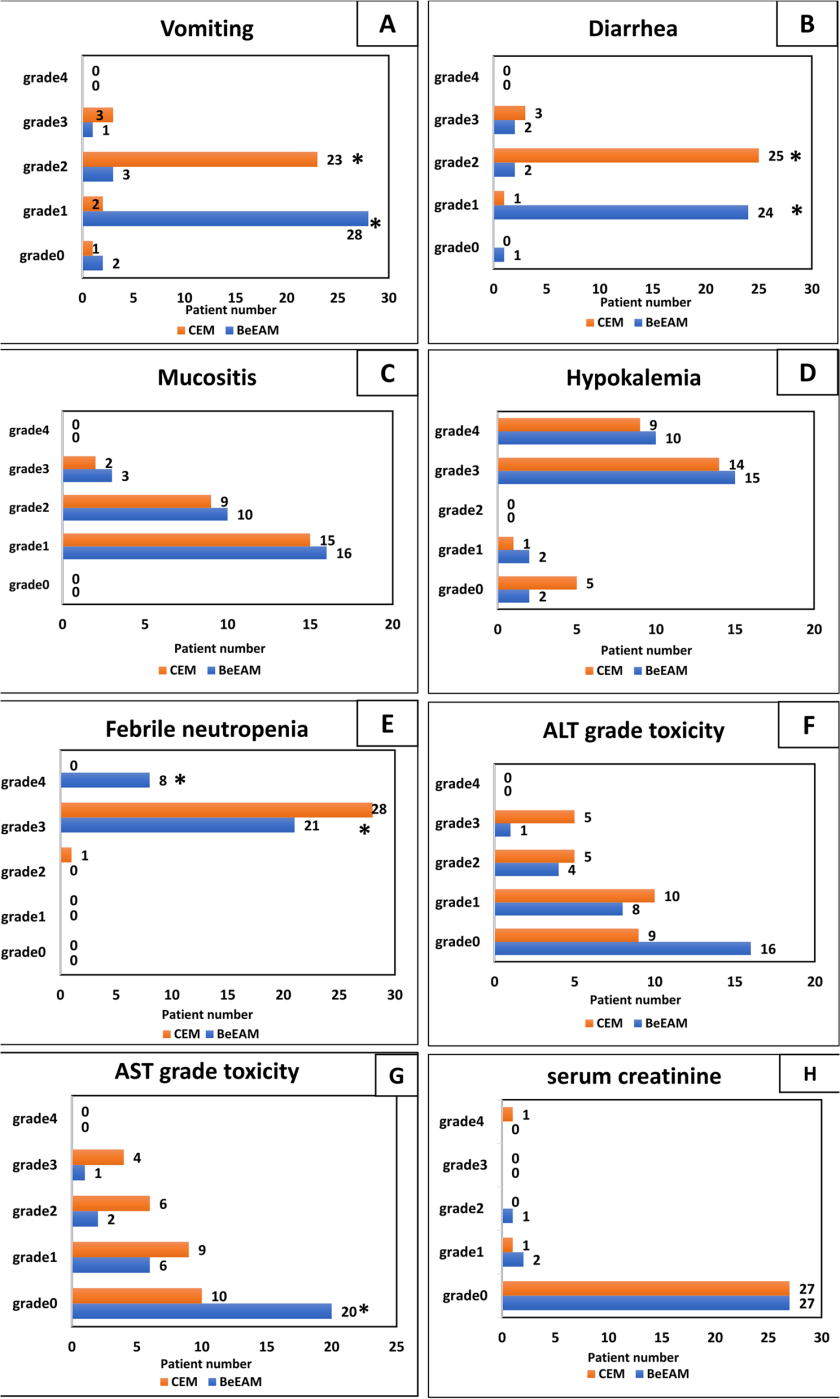
Adverse effects & related toxicities. **A** In CEM, vomiting grade 1 was observed in 2 patients, significantly fewer than in BeEAM where it was observed in 28 patients (*p* < 0.0001). Similarly, vomiting grade 2 occurred in 3 CEM patients compared to 23 BeEAM patients (*p* < 0.0001). **B**: Diarrhea grade 1 was less prevalent in CEM with 1 patient compared to 24 in BeEAM (*p* < 0.0001), while grade 2 was more common in CEM with 25 patients versus 2 in BeEAM (*p* < 0.0001). **C**: Mucositis. **D**: Hypokalemia. **E**: Febrile neutropenia grade 3 was higher in CEM (28 patients) than in BeEAM (21 patients) (*p* < 0.025), whereas grade 4 was absent in CEM but present in 8 BeEAM patients (*p* = 0.004). **F**: Alanine aminotransferase (ALT). **G**: Aspartate aminotransferase (AST) grade 0 (no elevated enzyme) was observed in 10 CEM patients compared to 20 in BeEAM. **H**: Serum creatinine. *: Mean there is a statistically significant difference

Both the CEM and BeEAM regimens were associated with adverse effects and related toxicities. All data are summarized in Fig. 4.

### Gastrointestinal toxicities

The CEM regimen showed a significant reduction in the percentage of patients experiencing chemotherapy-induced vomiting, for grade 0 (no vomiting), there was 1 patient in the CEM group compared to 2 patients in the BeEAM group, for grade 1 (2 patients vs. 28 in BeEAM, *p* < 0.0001), for grade 2 (3 patients vs. 23 in BeEAM, *p* < 0.0001), for grade 3 in CEM 3 patients vs 1 in BeEAM) and there were no grade 4 vomiting events in either group. This suggests the CEM regimen may have a more favorable antiemetic profile. Conversely chemotherapy-induced diarrhea, no patients in the CEM group experienced grade 0 diarrhea, meaning all patients in the CEM group suffered from diarrhea of varying grades compared to 1 patient in BeEAM. Grade 1 diarrhea was less prevalent in CEM (1 patient) than in BeEAM (24 patients, *p* < 0.0001), while grade 2 diarrhea was more common in the CEM group (25 patients) compared to the BeEAM group (2 patients, *p* < 0.0001). Grade 3 was more common in CEM 3 patients vs 2 patients in BeEAM group. There were no grade 4 diarrhea events in either group. This suggests the CEM regimen may have a more favorable gastrointestinal toxicity profile, may reduce vomiting, and it may be associated with a higher risk of severe diarrhea.

### Hematologic toxicities

Regarding febrile neutropenia, no grade 0 or grade 1 febrile neutropenia events were observed in either treatment group. For grade 2 febrile neutropenia, 1 patient experienced this event in the CEM group compared to none in the BeEAM group. However, grade 3 febrile neutropenia was more common in the CEM cohort, occurring in 28 patients versus 21 in the BeEAM arm (*p* < 0.025). Importantly, grade 4 febrile neutropenia was completely absent in the CEM regimen but was present in 8 patients receiving the BeEAM conditioning (*p* = 0.004). These findings suggest the CEM regimen may be associated with a lower risk of the most severe hematologic toxicities than the BeEAM approach. This is further supported by the results in Tables 3 & 4 which demonstrate faster hematologic recovery and reduced transfusion requirements with the CEM conditioning.

### Hepatic toxicities

Alanine aminotransferase (ALT) grade Toxicity:

- A higher proportion of CEM patients experienced elevated ALT levels across the toxicity grades compared to BeEAM. For grade 0 (no toxicity) 9 CEM patients versus 16 in BeEAM. For grade 1 (mild) toxicity, 10 CEM patients had increased ALT versus 5 in BeEAM. Grade 3 (severe) ALT toxicity was more common in CEM, with 5 patients affected versus 1 in BeEAM. No grade 4 (life-threatening) ALT toxicity was reported in either group.

Aspartate aminotransferase (AST) grade Toxicity:

- Grade 0 (no toxicity), 10 CEM patients had normal AST levels, while 20 BeEAM patients had no AST elevation, suggesting a greater proportion of BeEAM patients maintained normal AST levels. With statistically significant differences *p*=0.017
- At grade 1 (mild) toxicity, 9 CEM patients had increased AST, while there were 0 in BeEAM patients.
- Moderate grade 2 AST toxicity was more prevalent in CEM, with 6 patients affected versus 2 in BeEAM. Severe grade 3 AST toxicity was reported in 4 CEM patients but only 1 in BeEAM. No grade 4 (life-threatening) AST toxicity occurred in either treatment group.

In summary, the CEM regimen is associated with higher rates and severity of ALT and AST elevations versus the BeEAM regimen, especially at more severe toxicity grades. These differences in liver enzyme toxicity profiles warrant further investigation and discussion.

During the transplant process, there is typically a brief increase in ALT and AST levels two to three days following the conditioning regimen. Subsequently, there is a rapid decrease in these levels. We continuously monitor the enzyme levels and strive to minimize liver toxic agents while providing liver support as needed.

### Renal toxicities

Renal toxicity was closely monitored through serial assessment of serum creatinine (S.Cr) levels in both treatment groups. Regarding grade 0 renal toxicity (no S.Cr elevation), 27 patients in the CEM cohort and 27 patients in the BeEAM cohort experienced this outcome. For grade 1 renal toxicity, 1 patient in the CEM group and 2 patients in the BeEAM group were affected grade 2 renal toxicity events were observed in the CEM arm, whereas 1 patient in the BeEAM arm experienced this level of renal impairment. Importantly, there were no grade 3 renal toxicity events in either the CEM or BeEAM treatment groups. Concerning the most severe grade 4 renal toxicity, 1 patient in the CEM group was affected compared to 0 patients in the BeEAM group.

These results suggest the CEM and BeEAM conditioning regimens had a generally favorable renal safety profile, with most patients in both groups experiencing no significant renal toxicity. The observed grade 4 renal toxicity event in the CEM arm warrants further investigation, but the overall data indicate comparable renal safety between the two treatment approaches.

### Other toxicities

No significant differences were observed between the CEM and BeEAM regimens for other toxicities, such as oral mucositis and hypokalemia.

In conclusion, the CEM regimen displayed a varied toxicity profile compared to the BeEAM regimen. The CEM regimen resulted in significantly less chemotherapy-induced vomiting but had a higher incidence of severe diarrhea. Hematologic toxicities suggest the CEM regimen may be associated with faster hematologic recovery and fewer transfusion support needs than the BeEAM regimen. Hepatic toxicities were more severe with the CEM regimen, showing higher rates and severity of ALT and AST elevations, particularly at severe toxicity grades. No significant differences were noted for other toxicities, such as oral mucositis and hypokalemia. These findings underscore the necessity for extensive large-scale investigations into the toxicity profiles of these regimens to optimize patient outcomes.

### Survival analysis

**Fig. 5:**
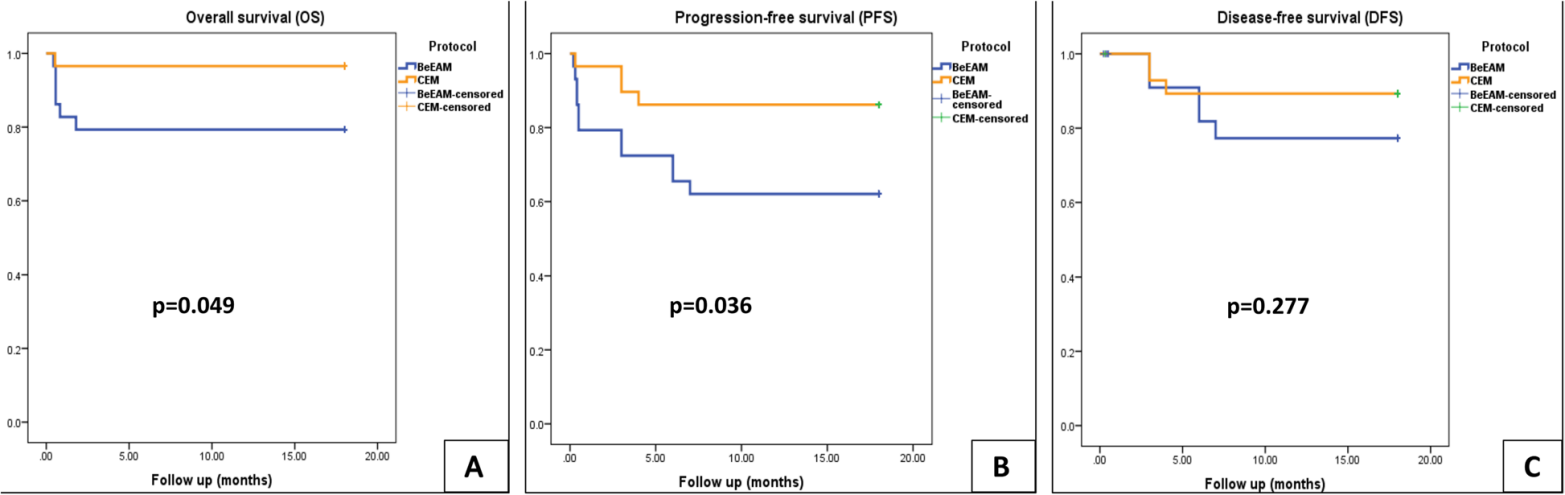
Survival analysis. **A** The Overall Survival (OS) curve demonstrated a significant difference between the two groups, as indicated by the log-rank test (*p* = 0.049), highlighting its statistical significance. **B** The Progression-Free Survival (PFS) curve also revealed a notable discrepancy between the groups, with a log-rank *p*-value of 0.036, indicating statistical significance. **C** The disease-free Survival (DFS) curve did not exhibit a significant variation between the groups, with a log-rank *p*-value of 0.277

During the 18-month follow-up period, the BeEAM protocol exhibited an Overall Survival (OS) probability of 79.31% (95% CI, 63.11–89.75%), whereas the CEM Protocol demonstrated a notably higher OS probability of 96.55% (95% CI, 84.91–99.44%). The log-rank test underscored a significant improvement in OS with the CEM regimen compared to BeEAM (*p* = 0.049). Similarly, Progression-Free Survival (PFS) was markedly enhanced in the CEM group (86.21%; 95% CI, 86.14–86.28%) vis-à-vis the BeEAM group (62.07%; 95% CI, 61.94–62.20%) with a statistically significant *p*-value of 0.036. However, the difference in Disease-Free Survival (DFS) between the two groups, although favoring CEM (82.76%; 95% CI, 82.67–87.85%) over BeEAM (89.66%; 95% CI, 85.20–94.12%), did not reach statistical significance (log-rank test *p* = 0.277), as depicted in Fig. 5. Furthermore, the BeEAM regimen evidenced a higher frequency of relapse (5 patients) compared to the CEM regimen (3 patients).

## Discussion

This study aimed to assess the efficacy and tolerability of the CEM protocol as a conditioning regimen for ASCT in lymphomas. An ideal conditioning regimen should demonstrate high efficacy while minimizing toxicity.

Previously, autologous transplantation for lymphoma without cryopreservation has been explored with standard BEAM regimens (carmustine, etoposide, cytarabine, and melphalan), where PBSCs stored at 4°C for up to 6 days were deemed safe for use in facilities lacking cryopreservation capabilities. However, results indicated delayed engraftment for both neutrophils and platelets, favoring cryopreservation availability due to decreasing PBSC viability over the regimen's duration [20].

Research suggests a viability window of up to 3 days for bone marrow stem cells, necessitating shorter-duration conditioning regimens to address this issue [17, 18].

In line with this, a retrospective study aimed to shorten conditioning regimen administration time to maintain viability, demonstrating that a non-cryopreserved approach may yield comparable or superior outcomes to cryopreserved strategies. The results showed that decrease in the occurrences of febrile neutropenia (60% vs. 37%, *P* = 0.008), reduced hospitalization durations (30 vs. 18 days, *P* = 0.001), accelerated engraftment (20 vs. 14 days, *P* = 0.001), and improved progression-free survival (72 vs. 44 months, *P* = 0.002) were observed. Furthermore, extended overall survival was noted with this approach [21].

Our findings support the usage of a condensed chemotherapy regimen administered over a short duration, such as the CEM protocol, to address the limited viability window of stem cells. The usage of CEM as a conditioning regimen is a suitable and logical conditioning regimen for bone marrow transplantation, as both etoposide and melphalan are components of many lymphoma protocols, including BeEAM and the standard BEAM protocol. The use of platinum-based chemotherapy, such as cisplatin, is common in protocols like DHAP, but carboplatin is considered safer. Carboplatin is also used in bone marrow transplantation for solid tumors, such as germ cell tumors and neuroblastoma, as previously mentioned.

Moreover, CEM effectively achieves the primary goal of the study: administering a high-dose conditioning regimen in a short, safe, and efficient manner, ensuring cell viability. This efficiency has led to superior results compared to BeEAM in terms of rapid engraftment. Consequently, patients required fewer blood and platelet transfusions compared to BeEAM, had a lower incidence of febrile neutropenia, and experienced shorter hospital stays, reducing overall costs for the patient. While monitoring other side effects such as diarrhea, which was more noticeable with CEM compared to BeEAM, we also carefully tracked fluid charts and fluid balance to prevent dehydration. Another observed side effect was elevated liver enzymes in CEM patients, typically elevated within the first two to three days and then decreased. we managed drug-induced liver injury, particularly from fluconazole, which is used as prophylaxis for neutropenia until recovered, post-market studies have shown it to increase transaminase levels [47, 48], We avoided its use until the enzyme levels peaked and patients approached neutropenia. We also employed liver support measures to mitigate this side effect. other toxicities, such as oral mucositis and hypokalemia, displayed no substantial distinctions between the two treatment approaches. Improved supportive care with the CEM regimen could enhance its outcomes, especially in the long term. The overall survival (OS), progression-free survival (PFS), and disease-free survival (DFS) rates were comparable, if not favorable, for CEM.

While our study provides meaningful insights into the clinical and safety outcomes of the BeEAM and CEM regimens, it is important to acknowledge certain limitations. One significant limitation is including an open-label nature and the relatively small sample size in each arm of the trial. Although our post-hoc power analysis using G*Power software version 3.1.0 indicated that a sample size of 58 patients would provide a power of 83.2% to detect a medium to large effect size of 0.80, the number of patients might still be insufficient to draw definitive conclusions. Given the small sample size, the results should be interpreted with caution. Further studies with larger cohorts are necessary to confirm these preliminary findings and to provide more robust and generalizable data. Nonetheless, the initial results are promising and suggest potential benefits of the CEM regimen in reducing certain adverse effects compared to the BeEAM regimen. Long-term follow-up studies are also recommended to assess the sustainability of these outcomes over time. By acknowledging this limitation, we emphasize the importance of continued research in this area to enhance the understanding and application of these conditioning regimens in lymphoma patients undergoing autologous hematopoietic cell transplantation.

## Conclusion

Our comprehensive investigation has revealed the distinct advantages of the CEM regimen over the BeEAM protocol in the context of ASCT for lymphomas. The CEM approach demonstrated enhanced rates of neutrophil and platelet recovery, shorter hospital stays, reduced incidence of adverse effects, lower treatment-related mortality (TRM), and decreased relapse rates. Furthermore, the CEM protocol conferred additional benefits in terms of overall survival (OS) and progression-free survival (PFS).

These robust findings suggest that the CEM regimen might hold significant promise as the preferred conditioning protocol for ASCT in lymphoma patients. Despite the promising results, it is important to acknowledge the inherent limitations of our study, particularly the modest sample size and limited follow-up duration. These factors suggest that while our findings are encouraging, they may not be definitive. The positive outcomes observed in this study should warrant further research with larger, more diverse patient cohorts and extended follow-up periods.

In summery, our study can highlight the potential of the CEM regimen as a preferred conditioning protocol for ASCT in lymphoma patients. Continued research and comprehensive evaluation in larger-scale studies are essential to confirm its efficacy, safety, and long-term clinical outcomes. Such efforts will ultimately assist evidence-based decision-making and optimize the care of lymphoma patients undergoing ASCT.

## Data Availability

The data are available from the corresponding author upon reasonable request. All data supporting the findings of this study are included within the paper, along with the original reference describing the microsatellites used in this study.

## Abbreviations

ABVD: Doxorubicin, Bleomycin, Vinblastine, Dacarbazine
ALT: Alanine aminotransferase
ASBMT: American Society for Blood and Marrow Transplantation
ASCO: American Society of Clinical Oncology
AST: Aspartate aminotransferase
BeEAM: Bendamustine, Etoposide, Cytarabine, Melphalan
BSA: Body Surface Area
CEM: Carboplatin, Etoposide, Melphalan
CR: Complete Remission
DHAP: Dexamethasone, Cisplatin, Cytarabine
DLBCL: Diffuse Large B-Cell Lymphoma
EBMT: European Society for Blood and Marrow Transplantation
ESHAP: Etoposide, Methylprednisolone, Cytarabine, Cisplatin
G-CSF: Granulocyte Colony-Stimulating Factor
GDP: Gemcitabine, Dexamethasone, Cisplatin
HCT: Hematopoietic Cell Transplantation
HDCT: High-Dose Chemotherapy
HL: Hodgkin's Lymphoma
HTN: Hypertension
IBW: Ideal Body Weight
ICE: Ifosfamide, Carboplatin, Etoposide
LEAM/CEAM: Lomustine, Etoposide, Cytarabine, Melphalan
MCL: Mantle Cell Lymphoma
MTD: Maximum-Tolerated Dose
NHL: Non-Hodgkin Lymphoma
OS: Overall Survival
PCNSL: Primary Central Nervous System Lymphoma
PFS: Progression-Free Survival
R-CHOP: Rituximab, Cyclophosphamide, Doxorubicin, Vincristine, Prednisone
R-DHAP: Rituximab, Dexamethasone, Cisplatin, Cytarabine
R-GDP: Rituximab, Gemcitabine, Dexamethasone, Cisplatin
R-ICE: Rituximab, Ifosfamide, Carboplatin, Etoposide
R-ESHAP: Rituximab, Etoposide, Methylprednisolone, Cytarabine, Cisplatin
R-Hyper-CVAD: Rituximab, Cyclophosphamide, Vincristine, Doxorubicin, Dexamethasone
TBW: Total Body Weight
TEAM: Thiotepa, Etoposide, Cytarabine, Melphalan
TRM: Transplant-Related Mortality
